# Thrombus composition in ischaemic stroke: histological and radiological evaluation, and implications for acute clinical management

**DOI:** 10.1007/s11239-025-03074-6

**Published:** 2025-03-21

**Authors:** Esmee Dohle, Abhishekh H. Ashok, Shiv Bhakta, Isuru Induruwa, Nicholas R. Evans

**Affiliations:** 1 Medical Sciences Division, John Radcliffe Hospital, University of Oxford, Oxford, UK; 2 Department of Radiology, University of Cambridge, Cambridge, UK; 3 Department of Clinical Neurosciences, University of Cambridge, Cambridge, UK; 4 Department of Stroke Medicine, Cambridge University Hospitals NHS Foundation Trust, Cambridge, UK

**Keywords:** Ischaemic stroke, Thrombosis, Histology, Radiology, Thrombectomy

## Abstract

Ischaemic stroke is one of the key causes of morbidity and mortality worldwide. Although rapid reperfusion through thrombolysis or mechanical thrombectomy is the cornerstone of acute management, the efficacy of these interventions is influenced by the underlying composition of the occluding thrombus, which varies widely. Histological examination of retrieved thrombi allows the determination of thrombus composition and may inform aetiology and secondary prevention strategies. Additionally, radiological features may provide valuable pre-treatment insights into thrombus composition to help predict treatment success. This narrative review discusses histological and radiological indicators of thrombus composition, and how this may predict success of thrombolysis and thrombectomy. Furthermore, it discusses how these insights can be applied in the diagnostic work-up of embolic stroke of undetermined source (ESUS), and the potential utility of emerging biomarkers relating to thrombus formation, in order to optimise secondary prevention strategies.

**Graphical Abstract:**

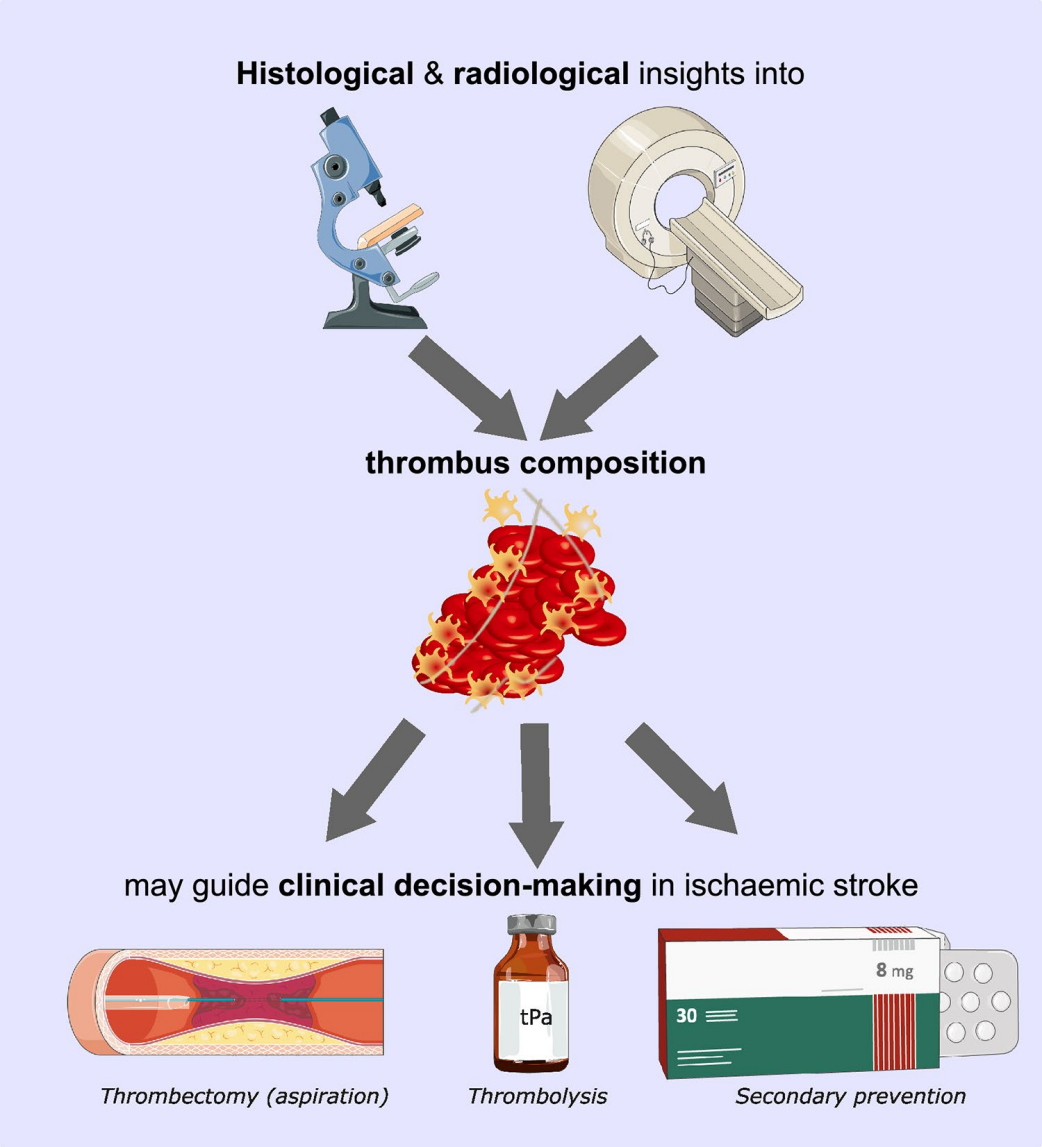

## Introduction

Globally, stroke is the second-leading cause of death and the third-leading cause of death and disability combined. [1] Changing demographics means that this burden is likely to rise: in the United Kingdom, the number of strokes is projected to increase by 60% between 2015 and 2035, with the number of stroke survivors more than doubling over the same period. [2] A similar rise in the worldwide incidence of stroke is also predicted. [3] This underscores the urgent need to optimize both hyperacute management and secondary prevention strategies to reduce the future burden of stroke.

Approximately 85% of all strokes are ischaemic strokes, where blockage of cerebral arteries leads to reduced blood supply and, if untreated, subsequent neuronal injury and cell death. [1, 4] The primary goal of hyperacute treatment is the swift restoration of cerebral perfusion, thereby minimising the extent of irreversible brain tissue loss and improving functional outcomes. Currently, the two mainstays of reperfusion treatment are intravenous thrombolysis using tissue plasminogen activator (tPA), and mechanical thrombectomy (MT); a neurointerventional procedure where the blood clot is physically extracted from the occluded vessel. Following acute management, the identification of index stroke aetiology aids in choosing appropriate secondary prevention to reduce the likelihood of stroke recurrence.

With the increasing use of mechanical thrombectomy in clinical practice, the ability to retrieve thrombi provides an opportunity for histopathological evaluation of thrombus composition. Additionally, advances in hyperacute stroke imaging, such as CT perfusion imaging, multiphase or delayed-phase CT angiography (CTA) and diffusion-weighted MRI, allow more detailed assessment of thrombus features, cerebral perfusion deficits, and overall tissue viability. Finally, blood biomarkers indicating inflammation, cardiac dysfunction, and endothelial injury are now more routinely available in acute clinical settings.

Faced with an individual with an acute ischaemic stroke, clinicians now have a much richer set of information at their disposal. This review uniquely synthesises information from histological studies, imaging studies and emerging blood biomarkers. We also discuss novel technologies advancing thrombus analysis, including radiomics, high-resolution scanning electron microscopy and quantitative PCR. We explore how these insights into thrombus composition and formation can be integrated to guide clinical decision-making both around hyperacute stroke management and secondary prevention.

## How does thrombus composition differ between stroke aetiologies?

The various aetiologies of acute ischaemic stroke reflect fundamentally different sites of thrombus formation. Major stroke subtypes, as delineated in the Trial of Org 10,172 in Acute Stroke Treatment (TOAST) classification, include embolic strokes secondary to large artery atherosclerosis and cardiogenic embolism. [5] The site of thrombus formation may influence the relative proportions and spatial distribution of the three primary clot components: red blood cells (RBCs), fibrin, and platelets. Other substantial components, including von Willebrand factor (vWF), leukocytes, neutrophil extracellular traps (NETs), and extracellular DNA are increasingly recognised. As a result of mechanical thrombectomy becoming a standard intervention for acute ischaemic stroke, [6–10] retrieved thrombi are now available for histopathological analysis, offering the potential for further insights into stroke aetiology [11, 12].

In the traditional view of the pathophysiology of clot formation within the heart, thrombus formation occurs secondary to damage to the endocardium and stasis within the left atrial appendage (often seen in cardiac diseases such as atrial fibrillation, valvular disease, or cardiomyopathies). Longer dwell times in the left atrial appendage enables progressive clot organization with increased accumulation and cross-linking of fibrin, as well as the incorporation of shattered or compressed red cells. Such clots are older, more stable, and more dense. In contrast, thrombus formation in large arteries occurs more rapidly and under high shear stress. Here, plasma lipoproteins, carrying an excess of cholesterol, penetrate sites with endothelial damage, triggering sub-endothelial collagen exposure and platelet activation and aggregation. Such agglutinative thrombi are torn away from the vessel wall soon after formation by turbulent and high-velocity blood flow, resulting in a looser, lesser-organised thrombus with fewer fibrin and more intact red cells [13–15].

The distinct thrombogenic processes and signatures according to stroke aetiologies is supported by several histopathologic studies and a recent systematic review suggesting a higher proportion and density of fibrin being present in cardioembolic thrombi. [11, 12, 16–22] This is supported by clinical studies highlighting the need for different anti-thrombotic strategies according to aetiology: antiplatelet therapies (with increasing use of dual-antiplatelet strategies) are the key pharmacological management for reducing recurrent large artery atherosclerosis strokes, [23] whereas anticoagulation has been shown to be superior to antiplatelets in reducing recurrent cardioembolic risk (with one large meta-analysis indicating a stroke risk reduction in atrial fibrillation of 64% and 22% with warfarin and antiplatelets respectively) [24]

The proportion of RBCs within a thrombus has also been extensively studied in relation to stroke aetiology. Several large-scale studies, the largest of which analysed clots from 1350 patients, reported higher proportions of intact RBCs in thrombi from large artery atherosclerosis compared to thrombi from a cardioembolic source [11, 12, 16–20, 25, 26] However, some smaller, older studies report contradictory findings, with higher proportions of RBCs in cardioembolic thrombi, [27–29] whilst other studies report no significant differences in RBC proportions between aetiologies at all. [30–34] One explanation for these disparate findings may be that clots undergo in situ ageing, reorganisation, and fibrin retraction post-retrieval, which may alter histological characteristics and complicate assessment of their composition.

The evidence for the constituent proportion of platelets in thrombi varying according to stroke aetiology is unconvincing. The majority of studies reporting no significant association between stroke aetiology and platelet proportion, [12, 17, 21, 32] whereas only one study identified a higher proportion of platelets in clots from a large artery source compared to a cardioembolic source. [33] However, several studies report that in clots from a large artery atherosclerosis source, platelets are distributed more peripherally, in contrast to the central clustering observed in cardioembolic clots. [17, 34] This difference in platelet distribution may be attributed to the distinct flow dynamics in each site: in large arteries, high shear forces push platelets towards the vessel wall, leading to peripheral distribution of platelets in clots. Conversely, in the heart, where flow is slower, platelets tend to aggregate centrally, forming clusters in the centre of the clot. [17, 34] An overview of key findings from histological studies regarding thrombus composition is shown in Table 1.

**Table 1:** Key findings regarding thrombus composition in histological studies

Key finding	Studies supporting this finding
Higher fibrin in cardioembolic thrombi	Liao et al. (2020) [11], Sporns et al. (2017) [12], Fitzgerald et al. (2021) [16], Ahn et al. (2016) [17], Maekawa et al. (2018) [18], Boeckh-Behrens et al. (2016) [19], Hund et al. (2023) [20], Huang et al. (2022) [21], Khismatullin et al. (2020) [22]
Higher RBCs in large artery thrombi	Liao et al. (2020) [11], Sporns et al. (2017) [12], Fitzgerald et al. (2021) [16], Ahn et al. (2016) [17], Maekawa et al. (2018) [18], Boeckh-Behrens et al. (2016) [19], Hund et al. (2023) [20], Brinjikji et al. (2021) [25], Niesten et al. (2014) [26]
Central platelet clustering in cardioembolic thrombi, peripheral platelet distribution in large artery thrombi	Ahn et al. (2016) [34], Kim et al. (2020) [34]

The contribution of other components, including neutrophils, vWF, NETs, and extracellular DNA, is increasingly recognised. Emerging evidence suggests that cardioembolic thrombi contain greater leukocyte, [11, 12] DNA, and NET content. [35, 36] This may be a result of the longer dwell times and blood stasis in the atria due to atrial fibrillation and low blood flow velocity. These conditions promote prolonged periods of thrombus formation, during which thrombus organization, fibrin accumulation, and fibrin cross-linking occur. [14]

Several histological techniques may be employed to analyse the composition of thrombi post-retrieval. Standard techniques such as haematoxylin and eosin (H&E) staining [12, 19] and Martius, Scarlet and Blue (MSB), which visualises fibrin, [16, 25] have been widely used in studies describing the proportion and distribution of red blood cells, fibrin and platelets. Specific proteins, such as von Willebrand factor, can be visualised with immunohistochemistry. [37] Advanced analysis methods have also been used, including high-resolution scanning electron microscopy for three-dimensional ultrastructural analysis [38, 39], and quantitative PCR or proteomics for detecting specific molecular markers such as NETs. [40, 41]

Understanding the relationship between clot composition and stroke aetiology is particularly useful for the diagnostic workup of an embolic stroke of undetermined source (ESUS). Evidence from the above histopathological studies suggest that thrombi in ESUS frequently have histologic features very similar to that of cardioembolic thrombi. [11, 12, 17, 19, 20] This suggests that many strokes of undetermined source are cardioembolic in aetiology, with the paroxysmal nature of some cases of atrial fibrillation limiting detection in the diagnostic work-up. Histological and radiological features that may aid in the workup of ESUS are discussed further below.

Importantly, it is worth noting an inherent limitation of all studies relying on histological examination of thrombi retrieved with mechanical thrombectomy: histological analysis is only possible on clots that have been successfully retrieved. This introduces a key bias, where the composition of resilient, un-extractable thrombi remains unknown. In the future, advanced imaging and molecular analysis methods may play a greater role in the examination of retrieved thrombi: techniques applied successfully so far include high-resolution scanning electron microscopy, [22] quantitative PCR analysis, [42] and gene ontology. [43]

These limitations highlight the potential utility of in vivo thrombus analysis, particularly through radiological assessment of clot characteristics. Not only would this allow analysis of thrombi that were unable to be aspirated, but may also have a role in prognostication and guiding treatment decisions in the acute setting.

## How does radiological analysis reflect clot composition and aetiology?

In a first episode of stroke, prior to histological information being available, radiological findings may provide clues to thrombus composition and the underlying aetiology. For instance, a sub-study from the MR CLEAN registry found that non-cardioembolic strokes (in comparison to cardioembolic strokes) are more commonly associated with specific radiological features including: [44]

- a hyperdense artery sign,
- a lower clot burden score (reflecting thrombus extent, from multisegment vessel occlusion to normal),
- a more proximal thrombus location,
- a shorter distance from the internal carotid artery terminus,
- higher absolute thrombus attenuation (indicating increased density on CT),
- longer thrombi.

**Fig. 1:**
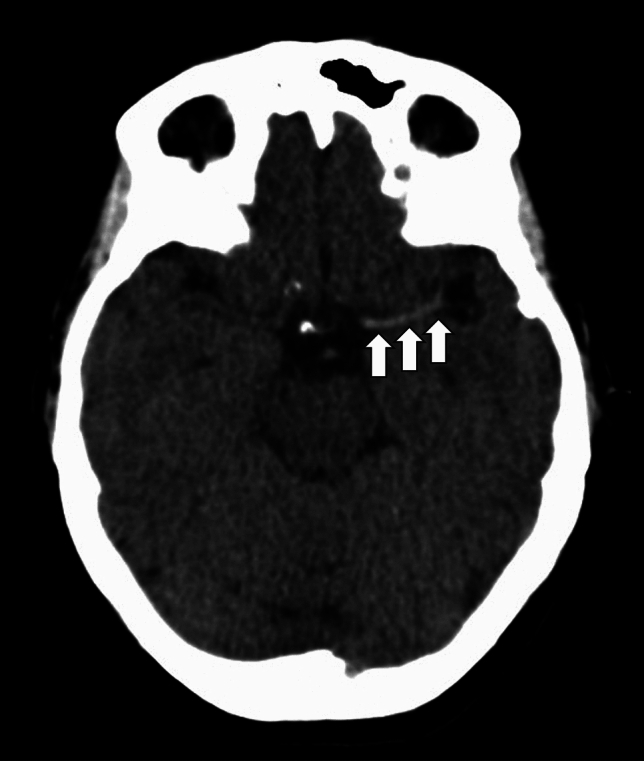
A non-contrast CT head demonstrating a hyperdense left middle cerebral artery indicative of thrombus (white arrows)

Interestingly, in addition to being associated with non-cardioembolic strokes, the hyperdense artery sign on non-contrast CT has also been shown to indicate RBC-rich thrombi. [30, 45] This corresponds with the previously discussed associations between non-cardioembolic strokes and RBC-rich thrombi, linking radiological findings to both stroke aetiology and thrombus composition. An example of the hyperdense artery sign is shown in Fig. 1. On gradient-echo MRI, a specialised MRI sequence which is particularly sensitive to magnetic components such as RBCs, the blooming artifact – where areas of clot appear larger than they actually are – has been associated with RBC-rich thrombi. [30] This occurs because the deoxyhaemoglobin and methaemoglobin content in RBCs disrupt the local magnetic field, leading to signal dropout and exaggerating the true size on final images.

In addition to specific signs, three radiological parameters are have been investigated to predict clot composition and provide insights into stroke aetiology: thrombus density, clot perviousness, and radiomic features. [46]

Assessment of ex vivo thrombi with D dual-energy CT, a technique which uses two different X-ray energy levels to differentiate tissue types, demonstrated that RBC-rich thrombi had higher clot attenuation in Hounsfield units, suggesting increased thrombus density, compared to fibrin/platelet-rich thrombi. [47] In a further study, higher attenuation on non-contrast CT was associated with RBC-rich thrombi, which were associated with a large artery atherosclerotic source. [26] Another study using microCT to assess retrieved clots found differential clot densities in RBC-rich, intermediate, and WBC-rich clots. [48]

Thrombus perviousness, assessed via single-phase or dynamic CTA, reflects the ease with which blood is able to flow through a clot and has been linked to thrombus composition and clinical outcomes following thrombectomy. [49–51] One retrospective analysis by Benson et al. reported that clot perviousness on CTA, defined as an increase of ≥ 10 Hounsfield units on CTA compared with NCCT, was associated with RBC-rich clots, whereas impervious clots were more likely to be fibrin- and WBC-rich. [50] However, some studies contradicted these findings, showing that pervious clots were higher in fibrin/platelet content. [52, 53] This discrepancy may arise from the heterogeneous nature of clots, which could have influenced the reference region chosen for perviousness measurement in these studies.

The increased use of machine learning and radiomics in acute stroke imaging has the potential to be exploited for thrombus evaluation. Several radiomic features have shown correlations with red blood cell, fibrin, and platelet content in thrombi, offering a more reliable prediction of cardioembolic stroke than routine radiological parameters. [54] A recent systematic review highlighted the clinical utility of radiomic measures and clot imaging, suggesting that automatic detection and segmentation of clots are essential for clinical integration. [55] Further methodological evaluation of predictive radiomics models is needed.

Other imaging modalities also offer future potential for in vivo thrombus evaluation. For instance, a preclinical study using diffuse reflectance spectroscopy (DRS) in a porcine model showed that DRS could reliably determine clot composition in vivo. [56] Positron-emission tomography (PET) is a highly sensitive imaging technique that uses radiotracers to detect pathophysiology of interest in vivo, [57, 58] and a number of emerging radiotracers have been developed specific to different thrombus components that may facilitate thrombus detection and evaluation. [59–63]

## How do histological and radiological factors affect efficacy of thrombolysis and mechanical thrombectomy?

The mainstays of hyperacute treatment in acute ischaemic stroke involve reperfusion strategies, either through systemic thrombolysis, where fibrinolytic agents such as alteplase are administered, or mechanical thrombectomy, which involves physical retrieval of thrombus from the affected artery. The clinical applicability of these treatments is patient-specific and governed by distinct criteria, which have been updated over time in response to emerging evidence. [64] Histological and radiological features may provide clues to thrombus composition and the likely efficacy of thrombolysis and mechanical thrombectomy.

As previously discussed, histological analysis of retrieved thrombi has provided key information about clot composition, which may affect treatment responsiveness to thrombolysis and thrombectomy. In particular, clots with higher RBC content have been found to be more responsive to thrombolysis with tissue plasminogen activator (tPA) both in clinical studies [65] and external thrombolysis attempts with retrieved human thrombi. [66] In the latter study, it was demonstrated that the low responsiveness to tPA in fibrin-rich, RBC-poor thrombi may be overcome by targeting non-fibrin components, e.g. with N,N-diacetyl-L-cystine or DNase-1.

Several considerations may explain the increased susceptibility of RBC-rich thrombi to thrombolysis, compared to fibrin-rich thrombi. It has been suggested that the presence of large amounts of intact RBCs may disrupt the gross fibrin network structure, leading to looser clot architecture. [67] Additionally, older work has shown that fibrin pores enlarge in the presence of RBCs, thereby increasing permeability and allowing improved access of thrombolytic agents [68].

Similar to thrombolysis, the treatment response to thrombectomy also appears to be affected by thrombus composition. Thrombi rich in RBCs are associated with a higher likelihood of successful recanalization and improved clinical outcomes following mechanical thrombectomy. [29, 69, 70] The reasons for this variation in treatment responsiveness are again thought to be related to thrombus composition. RBCs, where water is the main component, are more viscous, whereas fibrin is more elastic. [67, 71] Decreased stiffness of RBC-rich clots may improve clot-device interaction by allowing thrombectomy devices to more easily grasp and retrieve the thrombi. One study simulating stent retriever manipulation on thrombi of varying consistencies showed that fibrin-rich clots have higher friction coefficients with the vessel wall, making these clots more adherent to the vessel wall and therefore more resistant to device traction. [72] Interestingly, these friction coefficients rapidly increased following repeated stent retriever manipulation, echoing clinical experience that multiple attempts make resilient thrombi more sticky and less responsive to subsequent thrombectomy attempts. [72] However, the increased fragility of RBC-rich clots may have a downside: recent data suggests that they are more prone to fragmentation, leading to an increased risk of secondary distal embolization and necessitating proximal flow arrest with balloon catheters prior to attempting retrieval. [73] Further insights into the differential properties of thrombi with different compositions may lead to more targeted and individualised approaches to thrombectomy, both in terms of strategy and device choice.

Finally, it is worth noting that other key components of thrombi, including WBCs, platelets, extracellular DNA, and NETs may also affect the efficacy of reperfusion therapies. For instance, in a rat model, platelet-rich thrombi were shown to be more resistant to tPA. [74] Similarly, platelet-rich clots were shown to be associated with poorer recanalization rates following mechanical thrombectomy, [75] and it has been suggested that pre-treatment with antiplatelet therapy or vWF inhibition via targeting the glycoprotein Ib-vWF interaction could improve the efficacy of mechanical thrombectomy in such scenarios. [71] Additionally, a higher percentage of WBCs in a thrombus was associated with extended time to recanalization and poorer neurological recovery post-mechanical thrombectomy. [76]

Prior to histopathological information being available, radiological features may provide clues to the success of reperfusion therapy. For instance, a meta-analysis of twelve studies demonstrated that the hyperdense artery sign on non-contrast CT imaging is significantly associated with successful recanalization in patients who underwent mechanical thrombectomy. [77] However, no association was shown with clinical outcome. [77]

Thrombus perviousness, assessed via single-phase CTA, has been shown to predict clinical outcomes. [51] A subsequent study suggested that multiphase CTA, which captures temporal changes in blood flow, could be a better predictor of clinical outcomes following reperfusion therapy. [49] Thrombus perviousness was also linked to successful recanalisation following intravenous tPA treatment. [78] However, one study found that thrombus perviousness measured by multiphase CTA did not predict first-pass recanalisation or successful revascularisation in patients treated with a stent retriever. [79] Data from the COMPASS trial further indicated that clot perviousness was associated with first-pass aspiration success. [80]

**Fig. 2:**
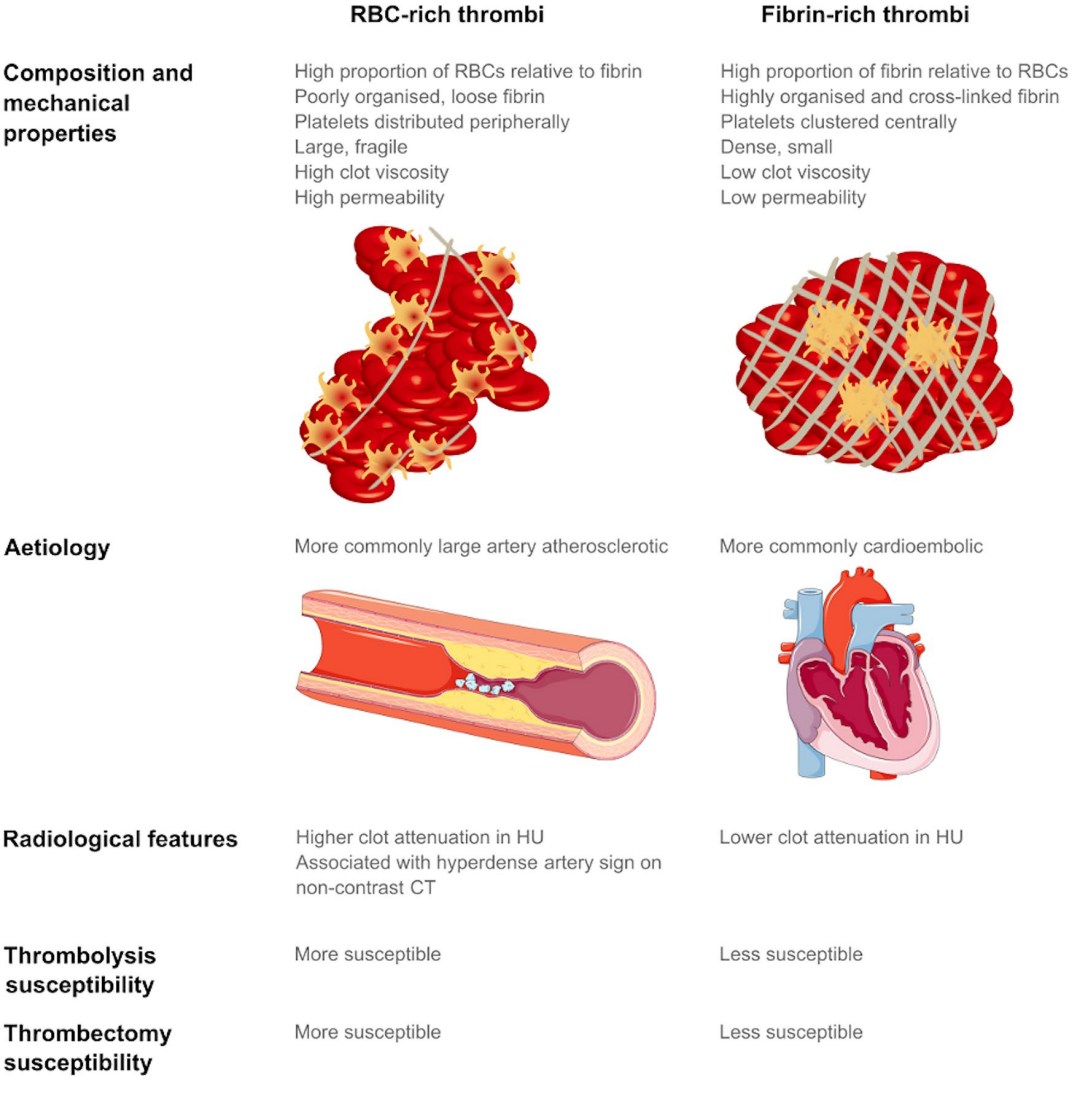
Comparison of RBC-rich thrombi and fibrin-rich thrombi

The radiological approach to thrombus composition continues to evolve. A nested case–control study of 67 patients with a large vessel occlusion assessed 326 predefined radiomics features that describe thrombus characteristics, such as first-order statistics, shape, size, and high-order statistical textural features. It used these radiomic features to train a linear support vector machine classifier, a commonly used supervised machine learning algorithm, to successfully predict successful recanalization with intravenous alteplase. [81] Another study identified nine radiomic features, reflecting clot heterogeneity, coarseness and texture in varying ways, that were predictive of first-attempt recanalisation with thromboaspiration. [82] Larger studies in radiomics and clot composition are needed to clarify their utility. [56] An overview of histological, radiological and reperfusion features of RBC-rich and fibrin-rich clots is shown in Fig. 2.

## How may clot analysis guide the work-up and secondary prevention of embolic stroke of undetermined source?

Evaluation of the thrombus – either through histological or radiological means – may have implications beyond acute treatment efficacy, such as improved diagnostics to guide secondary prevention. Selecting the appropriate secondary prevention strategy requires an understanding of the underlying aetiology in the individual patient. [83] As per the Trial of Org 10,172 in Acute Stroke Treatment (TOAST) criteria, embolic strokes of undetermined source (ESUS, sometimes called cryptogenic) are non-lacunar strokes where no underlying aetiology is found after evaluation, or where multiple potential aetiologies are present. [5] Approximately 25% of ischaemic strokes are cryptogenic, [84] and thrombus analysis to help determine the underlying aetiology has the potential to improve secondary prevention measures.

The cohort of ESUS patients is a heterogeneous one, encompassing numerous potential underlying aetiologies, [85] and characterised by a significant risk of recurrence. [86] Evaluation of patients with an unclear aetiology of ischaemic stroke may include continuous heart rhythm monitoring, [87] transthoracic echocardiography, [87] and screening for malignancy or thrombotic tendency, [88] along with multiple other potential investigations. [88, 89] Yet despite these investigations, which can be costly and can be delayed by availability, it remains common that no source for the stroke is identified. Hence, radiological or histological examination of the thrombus may provide valuable insights into the likely stroke aetiology.

**Fig. 3:**
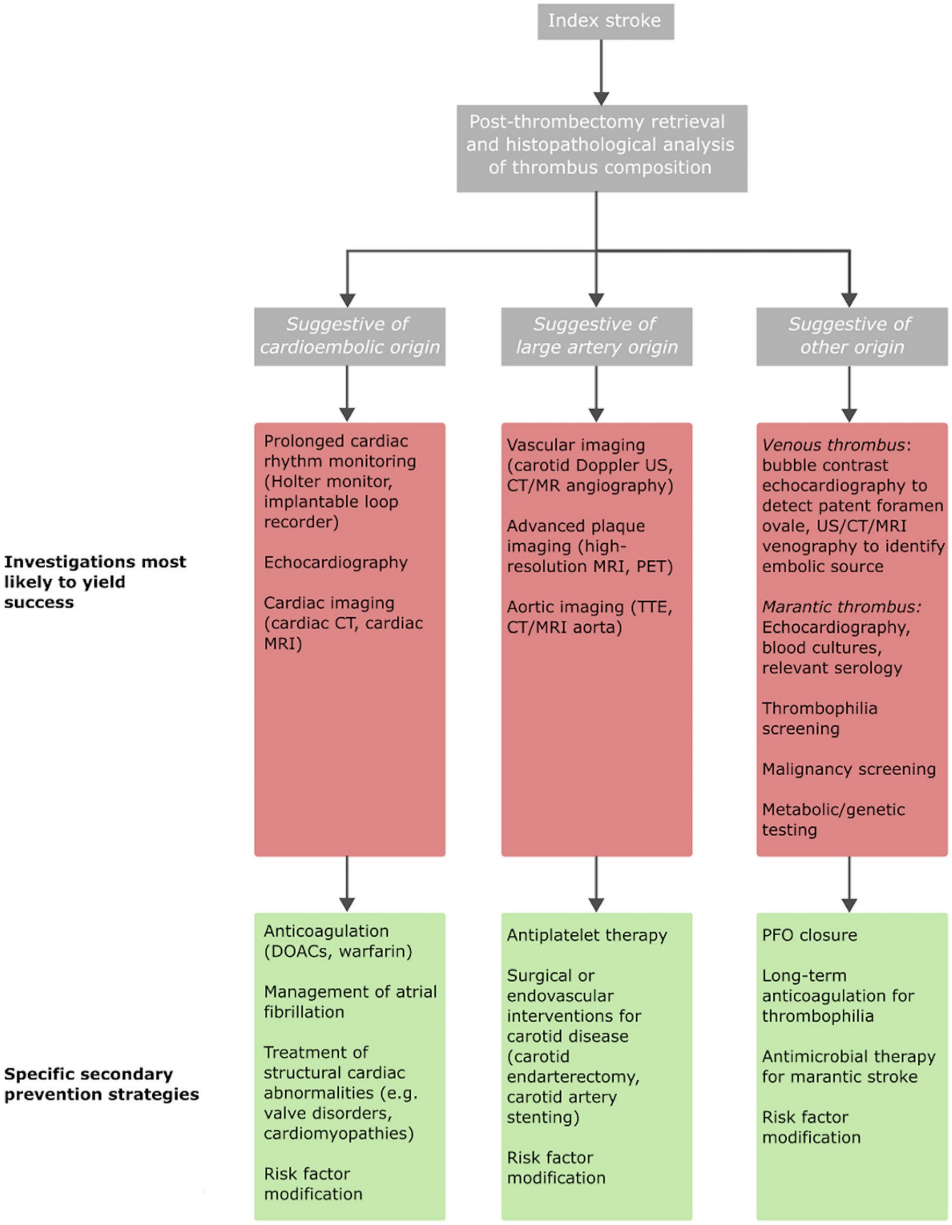
Flowchart of investigations and secondary prevention strategies based on thrombus composition analysis. Abbreviations: DOACs (Direct Oral Anticoagulants), CT (Computed Tomography), MRI (Magnetic Resonance Imaging), PET (Positron Emission Tomography), TTE (Transthoracic Echocardiography), US (Ultrasound), PFO (Patent Foramen Ovale)

Given the heterogeneity of ESUS aetiologies, the optimal treatment of ESUS remains unclear. Reflecting a hypothesis that ESUS likely reflects paroxysmal atrial fibrillation that has not yet been detected, a number of studies have investigated empirical anticoagulation in ESUS cohorts. However, the failure of empirical anticoagulation to demonstrate superiority over aspirin in trials such as ATTICUS and RE-SPECT ESUS highlights the challenges posed by this heterogeneity. [90–92] It is likely that subgroups within the ESUS trial population – such as those with non-stenotic atherosclerosis – may not benefit from anticoagulation, thereby diluting the overall treatment effect. Thrombus composition analysis therefore has the potential to refine patient stratification for trials. In a clinical context, thrombus analysis may provide the ability to target investigations more appropriately, improve their diagnostic yield, and reduce waiting times for investigations in order to provide more timely diagnoses and initiation of appropriate secondary prevention. An overview of suggested investigations and subsequent secondary prevention strategies, based on thrombus composition analysis, is shown in Fig. 3.

### Thrombus analysis to guide management in the presence of competing stroke aetiologies

As well as instances where no cause has been identified, ESUS may also include ischaemic strokes where concurrent aetiologies co-exist, such as the presence of atrial fibrillation and carotid atherosclerosis within the same individual.

Causative large artery atherosclerosis tends to be diagnosed where there is extracranial carotid artery stenosis of ≥ 50%. However, non-stenotic but symptomatic carotid plaques, as well as plaques in the aorta [93–95] are also potential sources of thrombi. [96] Visualising the area from the aortic arch to the intracranial vessels, [83] and identification of high-risk plaque, features is therefore required. Magnetic resonance and computed tomography angiography, alone, or in combination with positron emission tomography, can identify high-risk plaque features [58, 97] and underlying processes, such as inflammation [98] and microcalcification. [99] These processes are also potential targets to reduce plaque destabilisation and stroke recurrence, [100–103] though the availability of advanced imaging techniques and their radiation exposure limits their widespread adoption for ESUS stroke work-ups. Consequently, more accessible thrombus evaluation may provide surrogate markers of active but sub-threshold atheroma that has gone undetected. In addition, the use of thrombus analysis to identify an atherosclerotic cause of stroke could be used in the research setting to better phenotype the aetiology of the stroke as an inclusion criterion in future trials regarding treatment of atherosclerosis (where mistaken inclusion of non-atherosclerotic aetiologies may dilute treatment effects) [104].

A substantial fraction of patients with ESUS have atrial fibrillation detected during long term follow up, [105] but the clinical significance of these episodes, and need for anticoagulation, is unclear, [86] and may relate to the burden of atrial fibrillation. [106] In addition, patients may have a concurrent potential cause for their stroke, [107] which may complicate diagnosis and management. Where thrombus analysis suggests a cardioembolic origin, identification of the location of the thrombus and the underlying aetiology are required, as these have implications for optimal secondary prevention. [83] Current investigation techniques, such as echocardiography and cardiac rhythm monitoring, have their limitations, including high demand leading to delays in investigation, while having a low diagnostic yield in a general ischaemic stroke population [108].

Using thrombus analysis to select patients for relevant investigations may reduce overall demand, while increasing diagnostic yield. Given the routine use of interval neuroimaging in these patients, [109] additional imaging could be included to streamline investigation of potential cardioembolic causes. An example is cardiac computed tomography (CT) imaging at the time of neuroimaging, which may help detect the presence of intra-cardiac thrombi, [110] and other risk factors. Identification of atrial fibrillation through prolonged cardiac rhythm monitoring may be more clinically relevant in this population.

### Can thrombus analysis guide investigations into rarer aetiologies of stroke?

Reliable identification of venous emboli would allow the prioritisation of investigations into their source and route into the arterial circulation. Currently, clinical and imaging features [111] are used to quantify the likelihood of a PFO-related stroke. Positive identification of a venous origin of a thrombus would alter the clinical relevance of the presence of a PFO, and the risk–benefit calculation regarding PFO closure. This group could potentially also provide an enriched population for screening for thrombophilia.

Analysis of thrombus contents may provide a tissue diagnosis in infectious causes of stroke, [112] allowing earlier definitive antimicrobial administration. Further analysis methods [54, 113, 114] may also provide insights as to underlying aetiologies, allowing the targeting of investigations, including screening for malignancy in those with tumour cells within the thrombus. The presence of specific features within the thrombus could also increase the yield of screening, such as with incorporated antibodies with syphilis or antiphospholipid syndrome, or leukocyte enzymes in the case of Fabry disease.

As discussed previously, histological analysis of thrombus composition can provide clues to the likely aetiology (Fig. 2). This can subsequently inform investigations and secondary prevention strategies, an overview of which is shown in Fig. 3.

## Future outlook: how could serum biomarkers inform aetiology and guide clinical decision-making?

As outlined above, the histological and radiological appearances of thrombi have the potential to support decision making in ischaemic stroke and advancements in large vessel occlusion identification and retrieval techniques will further our existing knowledge on thrombus architecture and radiological appearance in ischaemic stroke. However, these techniques are limited to instances where a large vessel occlusion is present, accounting for approximately 30% of all ischaemic stroke cases [115].

As a result, serum biomarkers, often inexpensive and accessible, have been studied as an option to support clinical decision making in stroke. Currently, more than 150 candidate stroke biomarkers have been identified in ischaemic stroke. [116] Although there is a lack of published literature which link serum biomarkers to thrombus structure and composition, can clinicians still use serum biomarkers to detect the presence of a large vessel occlusion and to predict stroke aetiology? [116].

### Detection of large vessel occlusion

NETs are large extracellularly placed nuclear and mitochondrial DNA scaffolds studded with cytotoxic histones and proteases, that are created when neutrophils become activated under inflammatory conditions. [117] NETs act as a scaffold for red blood cells, platelets, and pro-coagulant proteins [118] and as a result, are a major constituent of ischaemic stroke thrombi. [119] ELISA assays are available that can quantify levels of NETs, by using surrogate markers such as cell-free DNA (cfDNA), myeloperoxidase-histone complexes, DNase activity, and circulating citrullinated histone H3 (citH3). [120] As further NET- biomarkers are identified, clinicians may be able to use these to predict the presence of a large vessel occlusion in stroke patients.

Glial fibrillary acidic protein (GFAP), a protein present in the soma and end-feet of astrocytes, has been identified as a promising candidate for differentiating haemorrhagic from ischaemic stroke. [121] During the first 6 h after a stroke, the serum concentration of GFAP is not only significantly higher in haemorrhagic stroke patients compared to ischaemic stroke but is also correlated to haemorrhage volume, allowing better differentiation of stroke type during the earliest stages of clinical assessment. [122, 123] Interestingly, when GFAP is combined in an assay with other sensitive markers for ischaemic stroke, such as d-dimer it can be used as a sensitive predictor of the presence of large vessel occlusion [124].

### Prediction of stroke aetiology

Currently no specific blood biomarker has consistently demonstrated the ability to predict ischaemic stroke aetiology. For example, evidence suggests that inflammatory biomarkers like C-reactive protein (CRP), IL (interleukin)-6, IL-1b, TNF-a and D-dimer alongside cardiac biomarkers such as B-type natriuretic peptide (BNP) or its N-terminal fragment, NT-pro-BNP are all associated with cardioembolic stroke. [125, 126] However, CRP has also been implicated in large artery stroke due to atherothrombosis [126] and small vessel strokes have been demonstrated to be associated with IL-6. [127] Furthermore, IL-6 and -16 have also been detected in high levels in large vessel stroke, highlighting the complexity of using biomarkers alone for aetiology prediction. [128] This overlap in biomarkers is not unexpected. Patients who are at risk of ishcaemic stroke have multiple overlapping vascular risk factors that increase the risk of stroke from various pathologies, making it difficult to specify stroke aetiology using a single biomarker. Furthermore, inflammatory pathways associated with vascular diseases result in the activation of a complex cascade of mediators, leading to multiple biomarkers being detectable at high levels in the blood [129].

In summary, serum biomarkers have the potential to be a powerful tool in supporting decision making in stroke patients. As this field evolves, the cross over application of high-throughput omics technologies will continue to uncover the complex and comprehensive interactions between biomarkers, thrombus formation and composition, and stroke pathology. Future research will need to prospectively enrol larger cohorts of patients, particularly with ESUS, to identify candidate biomarkers and combine it with histological analysis of stroke thrombi and radiological data to comprehensively understand the mechanisms of thrombosis in stroke.

## Conclusion

The heterogeneous nature of ischaemic stroke aetiologies, tailored nature of secondary prevention strategies, and expanding provision of acute reperfusion therapies is increasingly reliant on precision-based decision-making. Radiological and histological evaluation of the thrombus – information already available in the clinical domain – has the potential to facilitate better phenotyping of strokes whilst informing prognostication and acute treatment strategies.
